# Intra-articular injection with Autologous Conditioned Plasma does not lead to a clinically relevant improvement of knee osteoarthritis: a prospective case series of 140 patients with 1-year follow-up

**DOI:** 10.1080/17453674.2020.1795366

**Published:** 2020-07-23

**Authors:** Jasmijn V Korpershoek, Lucienne A Vonk, Tommy S De Windt, Jon Admiraal, Esmee C Kester, Nienke Van Egmond, Daniël B F Saris, Roel J H Custers

**Affiliations:** a University Medical Center Utrecht, Utrecht, the Netherlands; b Mayo Clinic, Rochester, USA

## Abstract

Background and purpose — Platelet-rich plasma (PRP) is broadly used in the treatment of knee osteoarthritis, but clinical outcomes are highly variable. We evaluated the effectiveness of intra-articular injections with Autologous Conditioned Plasma (ACP), a commercially available form of platelet-rich plasma, in a tertiary referral center. Second, we aimed to identify which patient factors are associated with clinical outcome.

Patients and methods — 140 patients (158 knees) with knee osteoarthritis (Kellgren and Lawrence grade 0–4) were treated with 3 intra-articular injections of ACP. The Knee Injury and Osteoarthritis Outcome Score (KOOS), pain (Numeric Rating Scale; NRS), and general health (EuroQol 5 Dimensions; EQ5D) were assessed at baseline and 3, 6, and 12 months’ follow-up. The effect of sex, age, BMI, Kellgren and Lawrence grade, history of knee trauma, and baseline KOOS on clinical outcome at 6 and 12 months was determined using linear regression.

Results — Mean KOOS increased from 37 at baseline to 44 at 3 months, 45 at 6 months, and 43 at 12 months’ follow-up. Mean NRS-pain decreased from 6.2 at baseline to 5.3 at 3 months, 5.2 at 6 months, and 5.3 at 12 months. EQ5D did not change significantly. There were no predictors of clinical outcome.

Interpretation — ACP does not lead to a clinically relevant improvement (exceeding the minimal clinically important difference) in patients suffering from knee osteoarthritis. None of the investigated factors predicts clinical outcome.

Platelet-rich plasma (PRP) has emerged as a potential treatment for osteoarthritis (OA). High levels of growth factors and cytokines present in platelets stimulate production of cartilage extracellular matrix, proliferation of chondrocytes, and migration of chondrocytes in vitro (Fortier et al. 2011, Fice et al. 2019). The potential beneficial effect of PRP in OA, together with the lack of regulatory restrictions in the use of these minimally manipulated autologous products, has rushed the field forward. The efficacy of PRP for the treatment of OA in clinical trials varies between no clinically relevant effect and a strong analgesic effect (Sánchez et al. 2012, Patel et al. 2013, Filardo et al. 2015, Gobbi et al. 2015, Forogh et al. 2016, Cole et al. 2017, Lin et al. 2019).

The efficacy of a commercially available PRP, Autologous Conditioned Plasma (ACP, Arthrex GmbH, Munich, Germany) has been proven in the setting of RCTs (Cerza et al. 2012, Smith 2015, Cole et al. 2017), but effectiveness has not been investigated in daily clinical practice. Moreover, the effect of different patient factors on the clinical outcome after ACP treatment is unknown.

This prospective case series aims to assess the effectiveness of ACP in clinical practice and to investigate the effect of sex, age, BMI, radiographic OA grade (Kellgren and Lawrence), history of knee trauma, and baseline Knee Injury and Osteoarthritis Outcome Score (KOOS) on clinical outcome. Since there is no consensus on whether PRP is more effective in mild or advanced OA (Lana et al. 2016, Jubert et al. 2017, Burchard et al. 2019), we included patients with symptomatic OA of all grades. We hypothesize that treatment with ACP leads to clinically relevant improvement in KOOS_5_ and that clinical outcome can be predicted with any of the investigated patient factors.

## Patients and methods

### Study design and setting

This prospective case series includes patients treated with ACP in an academic hospital (University Medical Center Utrecht, the Netherlands) between March 2017 and October 2018. A minimal follow-up of 1 year was chosen, because the effect of ACP reaches its maximum between 6 and 12 months (Cerza et al. 2012, Filardo et al. 2013, Cole et al. 2017). Inclusion criteria were: first series of ACP, symptomatic OA (Kellgren and Lawrence grade 0 to 4), sufficient understanding of the Dutch language to fill in the questionnaires and written informed consent. Exclusion criteria were: less than 3 ACP injections and earlier treatment with ACP.

### Patients

**Figure 1.**
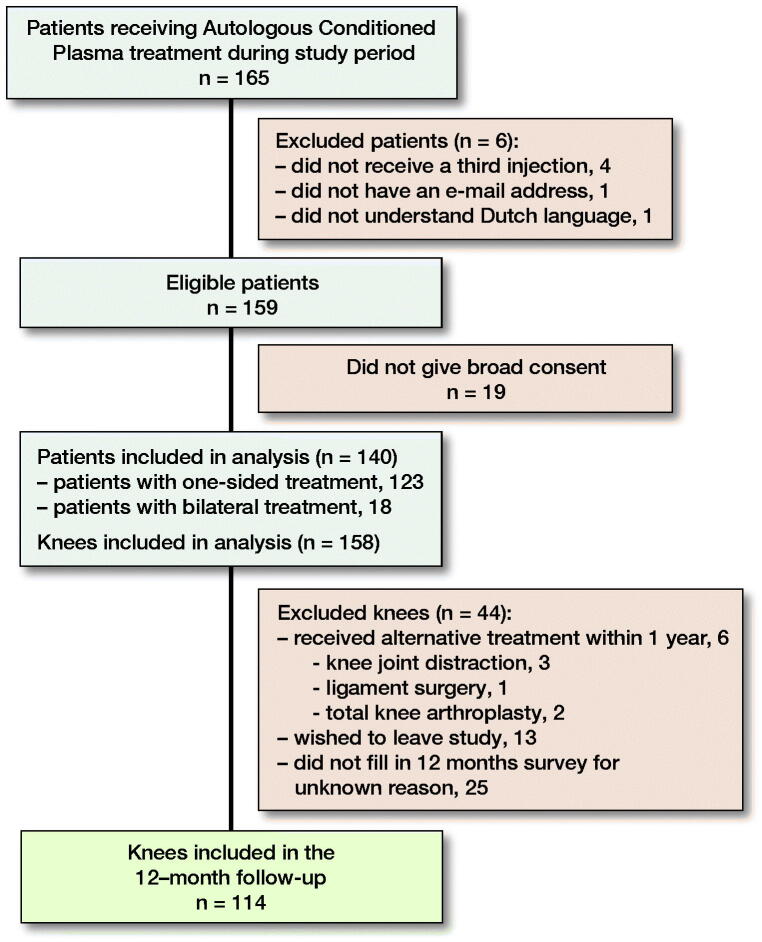
Patient recruitment.

140 patients (158 knees) could be included (Figure 1). 43 patients received 1 of the 3 injections with a 2-week interval (due to public holidays and other scheduling issues), all others received 3 consecutive injections with a 1-week interval. Sex, age, and BMI were collected from the patient records. History of knee trauma was defined as having a previous diagnosis of traumatic meniscus tear, cartilage defect or cruciate ligament tear. Baseline data were complete for all patient factors except BMI (35% missing) (Table 1). We did not monitor or correct for the use of other medications during the study period.

**Table 1.** Baseline characteristics of 140 included patients (158 knees)

Characteristic	N = 158
Age, mean (SD)	49 (10)
Female sex, n (%)	80 (51)
BMI, mean (SD)	28 (4.1)
History of traumatic injury, meniscus, anterior	
cruciate ligament, cartilage defect, n(%)	79 (50)
Baseline KOOS_5_, mean (SD)	37 (14)
Baseline NRS-pain, mean (SD)	62 (1.9)
Baseline EQ5D, mean (SD)	63 (19)
Bilateral treatment, n	18 (
Kellgren and Lawrence grade, n (%)	
0	8 (5.1)
1	40 (25)
2	55 (35)
3	43 (27)
4	12 (7.6)

Abbreviations: EQ5d, EuroQol 5 dimensions;

KOOS_5_, average of the 5 subscales of the Knee Injury and Osteo­arthritis Outcome Score (KOOS);

NRS pain, numeric rating scale;

SD, standard deviation.

### Radiographic assessment

Patients underwent anteroposterior and lateral view radiographies prior to treatment. Kellgren and Lawrence grade was assessed by 3 blinded observers. In any case where 1 observer rated the radiograph with 1 grade lower or higher than the others, the grade of the 2 observers was accepted. If the grades of 2 observers were 2 or more apart, agreement was reached in a consensus meeting. Interobserver reliability was assessed using a 2-way random intraclass correlation coefficient. The internal consistency of the Kellgren and Lawrence grade was good with a Cronbach’s alpha of 0.89.

### ACP preparation

The Arthrex ACP Double-Syringe System (Arthrex GmbH, Munich, Germany) was used for preparation of ACP. 15 mL of peripheral blood was drawn and centrifuged at 360G for 5 minutes to separate the blood components. Approximately 3–6 mL ACP was drawn into the inner syringe and injected into the knee joint using a superolateral approach with the patient in supine position.

### ACP composition

Using the CELL-DYN Emerald hematology analyzer (Abbott B.V., Abbott Park, IL, USA), 28 random samples of leftover material from ACP syringes were analyzed anonymously in order to characterize the administered PRP. Platelet, erythrocyte, and leucocyte concentration were measured in duplicate. The volume of injected material was documented.

### Patient reported outcome measures

Patients completed all questionnaires using an online survey tool (OnlinePROMS, InterActive Studios, Rosmalen, the Netherlands) at baseline and at 3, 6, and 12 months’ follow-up. Possible scores ranged from 0 to 100 (worst–best) for KOOS and EuroQol 5 Dimensions (EQ5D), and 0–10 (best–worst) for Numeric Rating Scale for pain (NRS pain). Dutch translations of KOOS (de Groot et al. 2008), EQ5D (EuroQol Research Foundation 2009), and NRS pain (LROI 2018) were used. In cases of bilateral treatment, patients filled in 2 separate surveys. Patients received a reminder after 5 and 10 days, and were contacted by telephone after 2 weeks in order to increase compliance. 89% of the patients filled out the survey at baseline, 87% at 3 months, 76% at 6 months and 75% at 12 months’ follow-up. Of patients who were lost to follow-up, data collected up to that point were included in the analyses.

### Data processing and statistics

Data were analyzed using IBM Statistical Package for the Social Sciences (SPSS) (version 15.0.0.2, IBM Corp, Armonk, NY, USA). Baseline patient factors are reported by means and standard deviation (SD) or number of patients and percentages. Outcomes are shown as average and 95% confidence intervals (CI). Missing data were not imputed; patients with missing outcome variables were not included in the analysis of those specific variables. P-values < 0.05 were considered significant.

The primary outcome, the effectiveness of ACP at 1 year, was evaluated using the change from baseline to 1-year follow-up in the average score on the 5 subscales of the KOOS (pain, symptoms, activities of daily living, sport and recreation, and knee-related quality of life). Change from baseline (ΔKOOS_5_) was estimated as an average population change using generalized estimating equations (GEE). ΔKOOS_5_ was compared with the minimal clinically important difference (MCID) recommended for KOOS (Roos 2020) using the CI. Since an MCID for non-operative OA treatment has not been defined and the MCID is highly variable based on calculation method and subscale of KOOS (Mills et al. 2016), we compare our data with the MCID of 8–10 recommended by the developers of the KOOS (Roos 2020).

In order to address selective loss to follow-up, using a subgroup analysis, patients lost to follow-up at 12 months were compared with the group that completed the follow-up. In another subgroup analysis, patients who returned for a second series of ACP injections after more than 1 year were compared with patients who did not undergo second ACP treatment. Baseline factors were compared between subgroups using t-tests for continuous variables and Pearson’s chi-square for quantitative variables.

Correlation between the ΔKOOS_5_ and sex, age, BMI, Kellgren and Lawrence grade, history of knee trauma, and baseline KOOS_5_ was assessed using GEE. As a rule of thumb, minimal sample size for a linear model is 10 patients per factor included in the model, therefore a minimum of 120 patients was included. Collinearity was assessed using correlation matrices, linearity using a scatterplot. Variables reaching a p-value lower than 0.2 in the univariate regression were entered in a multivariate regression model. Variables were removed from the multivariate model in order of p-value (highest first). Variables reaching a p < 0.05 in the multivariate model were retained.

### Ethics, funding, data-sharing, and potential conflicts of interest

This study was submitted to the institutional ethical review board of the University Medical Center Utrecht (METC 19-242, 03-04-2019; METC 17-005, 10-01-2017) and was conducted according to the World Medical Association Declaration of Helsinki. Written informed consent was obtained from all individual participants included in the study. This research was supported by the Dutch Arthritis Foundation (LLP-12). The study dataset is available from the corresponding author upon reasonable request. The authors declare that they have no competing interests.

## Results

### Patients (Figure 1)

Of all patients, 89% filled out the survey at baseline, 87% at 3 months, 76% at 6 months, and 75% at 12 months’ follow-up.

### ACP composition

Platelet concentration of 28 random anonymous samples of 18 patients was 513 (184)×10^9^/L, leucocyte concentration was 6.0 (10)×10^9^/L, and erythrocyte concentration was 0.07 (0.08)×10^9^/L. The average volume of the injected ACP from which these 28 samples were derived was 4.4 (0.8) mL.

### Patient-reported outcomes at 3, 6, and 12 months’ follow-up

**Figure 2.**
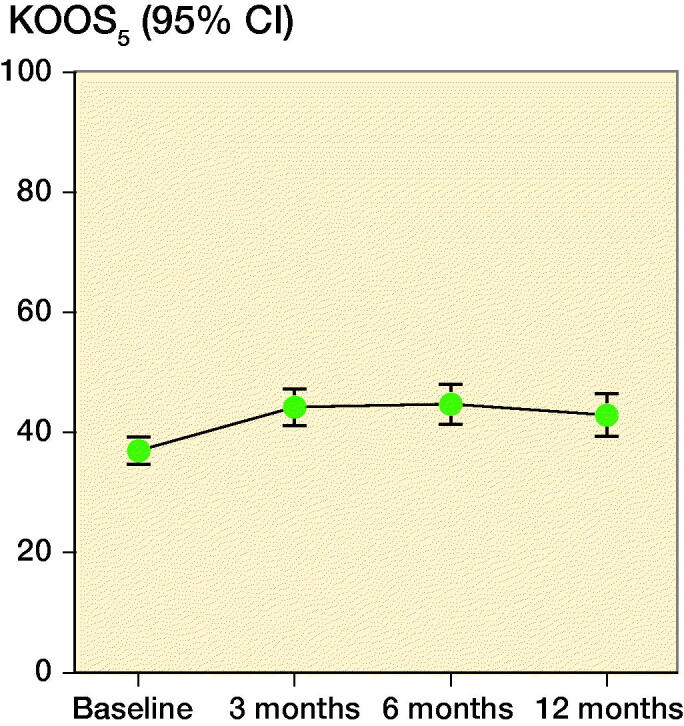
Mean (95% confidence interval) KOOS_5_ at baseline and after Autologous Conditioned Plasma treatment. KOOS_5_ is the average of the 5 subscales of the Knee Injury and Osteoarthritis Outcome Score (KOOS).

**Figure 3.**
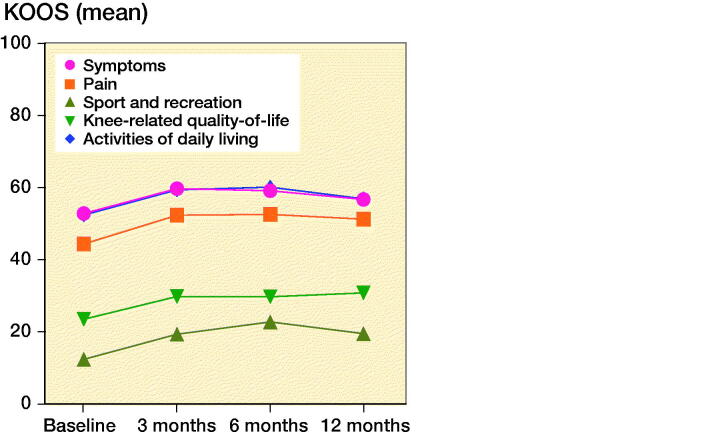
Mean Knee Injury and Osteoarthritis Outcome Score (KOOS) in the subscales pain, symptoms, function in activities of daily living, function in sport and recreation, and knee-related quality of life at baseline and after treatment with Autologous Conditioned Plasma.

Compared with baseline, KOOS_5_ increased at 3, 6, and 12 months after treatment (all p < 0.05; Figure 2). There were no statistically significant improvements between the follow-up-assessments. ΔKOOS_5_ partially overlapped with the MCID of 8–10 at 3 months (CI 4.9–9.5), 6 months (CI 4.7–11), and 12 months (CI 2.8–9.0) after treatment. At 6 months, 28% of patients reached the MCID of 8 or higher, 23% reached the MCID at 12 months. The change from baseline was comparable and statistically significant in all KOOS subscales (Figure 3). Pain (NRS) decreased from baseline to 3, 6, and 12 months after treatment, but did not improve statistically significant between follow-up assessments. EQ5D was similar in all of the assessments (Table 2).

**Table 2.** Patient-reported outcomes after treatment with Autologous Conditioned Plasma. Values are mean (confidence interval).

Scale	Baseline	3 months	6 months	12 months
KOOS_5_	37 (35–39)	44 (41–47)	45 (41–48)	43 (39–47)
EQ5D	63 (60–66)	64 (61–68)	67 (63–70)	66 (62–70)
NRS	6.2 (5.8–6.5)	5.3 (4.9–5.7)	5.2 (4.8–5.6)	5.3 (4.8–5.7)

### Loss to follow-up

At baseline, age, BMI, history of knee trauma, Kellgren and Lawrence grade, and KOOS_5_ of patients who were lost to follow-up at 12 months did not differ from patients who completed the follow-up. The group that was lost to follow-up consisted of more men (64%). At 3 months, patients who were lost to follow-up at 12 months had a ΔKOOS_5_ of 6.8 (CI 1.9–12), and patients who completed the follow-up had a ΔKOOS_5_ of 7.2 (CI 4.6–9.8). The missing values in KOOS_5_ at 12 months were imputed using the values of KOOS_5_ at 3 months in order to assess the effect of this loss to follow-up. The KOOS_5_ of the complete dataset, including the imputed data, is 43 (CI 40–46).

### Second series of ACP injections

After more than a year, a second series of ACP injections was given to 31 patients (34 knees). At baseline, these 31 patients did not differ from the others in sex, age, BMI, Kellgren and Lawrence grade, history of knee trauma, and KOOS_5_. At 6 months, the patients who later returned for a second series had a ΔKOOS_5_ of 15 (CI 9.4–21), whereas the patients who did not return for a second series of ACP injections had a ΔKOOS_5_ of 5.4 (CI 2.2–8.6). At 12 months, the patients who returned for a second series of injections had a ΔKOOS_5_ of 9.5 (CI 4.2–15), and the others had a ΔKOOS_5_ of 4.7 (CI 0.1–8.2).

### Linear regression

Sex, age, BMI, Kellgren and Lawrence grade, history of knee trauma, and baseline KOOS_5_ were not associated with clinical outcome (KOOS_5_) (Table 3). The variables sex, history of knee trauma, baseline KOOS_5_, and BMI were entered in a multivariate model, but not retained due to a p-value higher than 0.05.

**Table 3.** Univariate linear regression with coefficients of several factors in the prediction KOOS_5_

	Generalized estimating equations	
Factor	b (CI)	p-value
Age	–0.1 (–0.3 to 0.1)	0.4
Sex (male)	–4.0 (–8.6 to 0.7)	0.1
BMI	0.6 (–0.2 to 1.1)	0.1
History of traumatic injury ^a^	–0.5 (–5.1 to 4.1)	0.8
KOOS_5_ at baseline	–0.1 (–0.3 to 0)	0.1
Kellgren and Lawrence grade		0.1
0	Reference category	
1	–8.2 (–19 to 2.1)	0.1
2	–1.5 (–12 to 8.7)	0.8
3	–4.5 (–15 to 5.9)	0.4
4	0.4 (–13 to 13.1)	0.9

**^a^**Meniscus injury, anterior cruciate ligament rupture, cartilage defect

Abbreviations: CI, 95% confidence interval; KOOS_5_, average of the 5 subscales of the Knee Injury and Osteo­arthritis Outcome Score (KOOS).

## Discussion

In this prospective case series, treatment with intra-articular ACP for knee OA led to a statistically significant, but not clinically relevant, improvement of the KOOS_5_ after 3, 6, and 12 months’ follow-up. None of the investigated patient factors predicted clinical outcome, in contrast to our hypothesis. The highest change from baseline (ΔKOOS_5_) was observed at 6 months and did not exceed the MCID for KOOS (Roos 2020). In patients who returned for a second series of ACP injections after 1 year, the ΔKOOS_5_ exceeded the MCID at 6 months, but decreased at 12 months. 79% of patients did not return for a second series, due to a longer-lasting improvement, or, based on the low ΔKOOS_5_ in these patients at 6 months, more likely due to insufficient improvement.

Poor clinical results were described previously in an RCT using a different PRP composition (Di Martino et al. 2019). After treatment with PRP, no superior clinical improvement was found compared with hyaluronic acid and the improvement in IKDC score (International Knee Documentation Committee) did not reach the MCID (Irrgang et al. 2006). However, reported results of PRP treatment are predominantly good (Shen et al. 2017, Belk et al. 2020) and we expected a higher ΔKOOS_5_ after treatment.

An important source of variation and possible explanation for our findings is the different settings in which studies are executed. In an RCT, the efficacy of PRP is investigated under controlled circumstances. The participants are selected in order to minimize comorbidity and the protocol is designed to reach maximal patient and caregiver compliance. In this prospective case series, the effectiveness of PRP was investigated in the setting of daily clinical practice (Haynes 1999, Revicki and Frank 1999) and our real-world data show that ΔKOOS_5_ does not exceed the MCID. Moreover, the observed improvement might be largely attributable to a placebo effect, as a recent meta-analysis showed that placebo injections can lead to a clinical improvement above the MCID in RCTs (Previtali et al. 2020). The placebo effect in clinical practice might be even larger (Dieppe et al. 2016). Additionally, regression to the mean might contribute to the observed effect in our study, especially since the population is highly selected by inclusion from a tertiary referral center (Morton and Torgerson 2003). Furthermore, difficulty of publication of negative results, especially of non-randomized studies, might lead to publication bias, which is not considered in recently published meta-analyses (Shen et al. 2017, Belk et al. 2020). Lastly, differences in rehabilitation protocols, number of injections, varying composition between different preparations (Fitzpatrick et al. 2017), and administration intervals might influence clinical outcome. This remains a black box for PRP and hampers comparability of studies.

The poor results cannot be attributed to the composition of ACP, as the current composition is similar to that reported by Cole et al. (2017) and the manufacturer (Arthrex 2018), with approximately twice the platelet concentration of peripheral blood (Biino et al. 2013) and a leucocyte concentration classified as minimal (Delong et al. 2012). However, we found a notable variability in platelet and leucocyte concentration. In addition, we did not measure concentrations of cytokines and growth factors, which could provide useful information on the bioactivity of ACP.

Notable differences in patient populations do exist between our study and other ACP studies. We included patients in a tertiary referral center for joint preservation, with severe complaints and almost 10 points lower baseline KOOS compared with another ACP study (Filardo et al. 2013). This could mean that ACP is not effective in patients with severe complaints, even though our regression analysis indicated that baseline KOOS does not predict clinical outcome. Second, our patient age (mean 49 years) was lower than that in other ACP studies (mean 55–59 years) (Cerza et al. 2012, Filardo et al. 2013, Cole et al. 2017), but we found no effect of age on clinical outcome, similar to the results of Cole et al. (2017). In 2 studies (Filardo et al. 2011, 2013), younger age was even associated with a better outcome. Third, patients with post-traumatic OA were included in our series, while other studies have excluded patients with a history of knee surgery (Cerza et al. 2012) or treatment for a cartilage defect (Smith 2015), but in our case series history of knee trauma did not predict clinical outcome. Lastly, we included 18 patients with bilateral complaints, whereas these patients were excluded in other studies (Smith 2015, Cole et al. 2017). Patients with bilateral complaints have lower physical function and lower probability of improvement than patients with unilateral OA, and PROMs are influenced by contralateral knee pain (White et al. 2010, Riddle and Stratford 2013). To summarize, notable differences exist in patient population, but based on the results of our regression analysis and the small number of patients with bilateral complaints, these differences cannot fully explain our poor clinical outcome.

### Limitations

First, this is a prospective case series, thus lacking a control group. Since previous RCTs showed efficacy of ACP under ideal circumstances, we explicitly chose to investigate effectiveness in clinical practice. As a result, 43 patients received 1 of the intra-articular injections with a 2-week interval, while the others received all injections with a 1-week interval. This might result in variation in effectiveness, which is also a drawback for implementation of PRP in daily practice and could explain the differences between outcomes in RCTs and our clinical data. Second, within this heterogeneous patient population, various patient factors could influence clinical outcome, but limiting our exclusion criteria allowed us to study a population representative of the (heterogeneous) population in our clinical practice and to evaluate the influence of patient factors on treatment outcome. At the same time, the small number of included patients with Kellgren and Lawrence grade 0 and 4 limits generalizations in these groups. Effectiveness will need to be investigated in a larger cohort of patients with early (non-radiographic) or end-stage (grade 4) OA. Third, a relatively large patient group was lost to follow-up. However, the average KOOS_5_ did not change substantially when missing data at 12 months were imputed using data at 3 months. We therefore estimate the effect of this loss to follow-up to be small. Lastly, the MCID recommended for KOOS is 8–10 (Roos 2020), but the MCID in OA patients can actually range between 1.5 and 21 depending on calculation method and KOOS subscales (Mills et al. 2016), and does not account for the invasiveness of the treatment or its placebo effect. An MCID for non-invasive OA therapy should be established in order to determine whether the demonstrated effectiveness reaches a meaningful level for patients.

### Implications

There was no clinically relevant improvement in the majority of patients, nor did most patients return for additional ACP treatment. No predictors of improved clinical outcome were identified. In the limited number of patients who reached the MCID, the effect of ACP decreased between 6 and 12 months, necessitating a second series of treatment after 1 year. In our view, ACP should not be used in daily clinical practice in the current form and population. Future research should try to improve the clinical outcome of this treatment by optimization of the composition of PRP and/or patient selection, before implementation in daily practice. This study demonstrates the gap between efficacy in RCTs and effectiveness in clinical practice, which underlines the importance of evaluating effectiveness after market approval.
